# The Stemness of Human Ovarian Granulosa Cells and the Role of Resveratrol in the Differentiation of MSCs—A Review Based on Cellular and Molecular Knowledge

**DOI:** 10.3390/cells9061418

**Published:** 2020-06-07

**Authors:** Malgorzata Jozkowiak, Greg Hutchings, Maurycy Jankowski, Katarzyna Kulcenty, Paul Mozdziak, Bartosz Kempisty, Robert Z. Spaczynski, Hanna Piotrowska-Kempisty

**Affiliations:** 1 Department of Toxicology, Poznan University of Medical Sciences, Dojazd 30 St., PL-60-631 Poznan, Poland; malgorzata.jozkowiak@gmail.com; 2 Department of Anatomy, Poznan University of Medical Sciences, Swiecickiego 6 St., PL-60-781 Poznan, Poland; g.hutchings.16@abdn.ac.uk (G.H.); mjankowski@ump.edu.pl (M.J.); bkempisty@ump.edu.pl (B.K.); 3 Radiology Lab, Department of Medical Physics, Greater Poland Cancer Centre, Garbary 15 St., PL-61-866 Poznan, Poland; katarzyna.kulcenty@wco.pl; 4 Physiology Graduate Program, North Carolina State University, Campus Box 7608, Raleigh, NC 27695-7608, USA; pemozdzi@ncsu.edu; 5 Department of Histology and Embryology, Poznan University of Medical Sciences, Swiecickiego 6 St., PL-60-781 Poznan, Poland; 6 Department of Veterinary Surgery, Institute of Veterinary Medicine, Nicolaus Copernicus University in Torun, 87-100 Torun, Poland; 7 Department of Obstetrics and Gynecology, University Hospital and Masaryk University, 60200 Brno, Czech Republic; 8 Division of Infertility and Reproductive Endocrinology, Department of Gynecology, Obstetrics and Gynecological Oncology, Poznan University of Medical Sciences, Polna 33 St., PL-60-535 Poznan, Poland; rspaczynski@yahoo.com

**Keywords:** mesenchymal stem cells, granulosa cells, differentiation, resveratrol, SIRT1

## Abstract

Ovarian Granulosa Cells (GCs) are known to proliferate in the developing follicle and undergo several biochemical processes during folliculogenesis. They represent a multipotent cell population that has been differentiated to neuronal cells, chondrocytes, and osteoblasts in vitro. However, progression and maturation of GCs are accompanied by a reduction in their stemness. In the developing follicle, GCs communicate with the oocyte bidirectionally via gap junctions. Together with neighboring theca cells, they play a crucial role in steroidogenesis, particularly the production of estradiol, as well as progesterone following luteinization. Many signaling pathways are known to be important throughout the follicle development, leading either towards luteinization and release of the oocyte, or follicular atresia and apoptosis. These signaling pathways include cAMP, PI3K, SMAD, Hedgehog (HH), Hippo and Notch, which act together in a complex manner to control the maturation of GCs through regulation of key genes, from the primordial follicle to the luteal phase. Small molecules such as resveratrol, a phytoalexin found in grapes, peanuts and other dietary constituents, may be able to activate/inhibit these signaling pathways and thereby control physiological properties of GCs. This article reviews the current knowledge about granulosa stem cells, the signaling pathways driving their development and maturation, as well as biological activities of resveratrol and its properties as a pro-differentiation agent.

## 1. Introduction

Resveratrol, a naturally occurring polyphenol, was initially isolated from the roots of white hellebore (*Veratrum grandiflorum*). Then in 1964, resveratrol was identified as the active compound of the roots of *Polygonum cuspidatum*, traditional medicinal plant mostly used in Asia [1,2]. *Polygonum cuspidatum* is commonly known as a Japanese knotweed or Itadori plant. In Japanese, Itadori refers to “well-being” and Itadori tea has been broadly used to treat various diseases including atherosclerosis, skin inflammations, allergies and cardiovascular diseases [1]. Nowadays, as a result of growing interest in natural medicine, resveratrol has been described as a polyphenol component in over 70 kinds of plants belonging to 21 families and 31 genera, e.g., *Vitis L.*, a member of to *Vitaceae* family; *Arachis L.*, *Sophora L., Cassia L.* of the *Leguminosae* family, and *Eucalyptus I. Herit* (family *Myrtaceae*) [3]. In 1976, resveratrol was detected in grapes (*Vitis vinifera*), where it occurs in significant concentrations in leaf epidermis and skins but not the flesh [3,4,5]. Considering that grape skins are not fermented in the white wine production, only red wines contain considerable concentration of resveratrol, which fluctuates between 1.5–3.0 mg/L [3,5]. Regular consumption of moderate amounts of red wine is suggested as an explanation for the “French Paradox”, the term describing a relatively low risk of cardiovascular disease despite of high intake of dietary cholesterol and saturated fat [6]. However, it is of interest that one tablet of resveratrol supplement (e.g., 250 mg) is equivalent to more than hundred bottles of red wine.

Based on previously conducted studies, granulosa cells (GCs) are considered to possess stem cell characteristics [7,8]. They have been reported to transdifferentiate into osteoblasts and chondroblasts in the presence of leukaemia-inhibiting factor (LIF) or follicle stimulating hormone (FSH) [9,10,11]. It is well known that resveratrol influences the differentiation of stem cells into various cell lines. A recent investigation of Di Benedetto et al. showed that polydatin, precursor of resveratrol, promotes osteogenic differentiation of dental bud stem cells [12]. In the light of these findings, resveratrol and/or its analogs might also influence the differentiation of GCs.

Recently, the therapeutic potential of mesenchymal cells has been extensively studied. As GCs exhibit several mesenchymal-like characteristics, evaluation of new agents promoting their differentiation may both indicate them as a potential model for modern, advanced pharmacogenetics, but also make them more relevant to the current, clinical medicine.

Additionally, GCs seem to be a convenient and attainable source of stem cells since they are easy to collect from the ovarian follicles obtained during oocyte pickup from women undergoing assisted reproductive techniques.

Hence, this article reviews the current knowledge about granulosa stem cells, the signaling pathways driving their development and maturation, as well as biological activities of resveratrol, including its properties as a pro-differentiation agent.

## 2. Histological and Cellular Properties of Human Ovarian Granulosa Cells

The mammalian ovary holds the reservoir of gametes released throughout the course of the female fertility period. Furthermore, it produces hormones which control the menstrual cycle, ovulation and pregnancy [13]. Developing oocytes are reliant on the microenvironment formed by the surrounding somatic cell layers, as well as bidirectional signaling with the cells that constitute them. These include theca cells and GCs, which aid in folliculogenesis and oocyte maturation. GCs are vital to various processes in follicle development and the menstrual cycle, including steroidogenesis, with their innermost sub-population, the cumulus oophorus cells, communicating with the oocyte via gap junctions [14]. Theca cells produce androgens which are then aromatized into estradiol by the neighboring GCs during folliculogenesis [7].

Folliculogenesis begins as early as the embryonic development. Primordial germ cells (PGCs) form clusters eventually becoming the primordial follicle, a small, isolated space in the ovarian outer cortex surrounded by multiple layers of unique cells, in which a single oocyte will grow. At this stage, the epithelial layer surrounding the primordial follicle is composed of pre-GCs, which maintain direct contact with the oocyte until it begins growth. At this point, they differentiate into GCs, become cuboidal in shape and are separated from the oocyte by the zona pellucida (ZP) [15]. A change in GC cell shape from flattened to cuboidal marks the transition from primordial to primary follicle [16]. Cell shape regulates proliferation and steroidogenesis in GCs, with their proliferation and differentiation both occurring during folliculogenesis [17]. Assumption of cuboid shape by the GCs is dependent on the presence of adherens junctions and their associated proteins, β-catenin, N-cadherin and nectin 2 [18].

Development of blood vessel networks and angiogenesis during folliculogenesis are vital to granulosa cell function, providing oxygen and nutrients. The development of the follicle and oocyte are aided by GCs via paracrine and juxtracrine factors [19]. Several factors determine which of the primordial follicles will develop into primary, preantral and Graafian stages of development, a process governed largely by communication between the oocyte and surrounding somatic cells, including GCs. Follicles which are discarded undergo a process called follicular atresia, involving apoptosis of GCs [20]. More than 99.9% of human oocytes (in contrast to 75% of rodent oocytes) degenerate at some stage of development and never ovulate [21].

A subset of antral follicles, known as the dominant follicles, will progress to the pre-ovulatory stage in each menstrual cycle. In response to luteinizing hormone (LH), ovulation occurs and the oocyte is released, in tandem with the rupture of basal lamina, luteinization of theca and GCs, and formation of the corpus luteum (CL) [16,22]. CL expresses higher concentrations of steroidogenic enzymes (predominantly *P450scc*), leading to significant increase in pregnenolone and progesterone production. Granulosa lutein cells express aromatase and also produce estrogens. However, the predominant shift in progesterone production with distinct midluteal peak marks the progression to luteal phase [23].

GCs can be broadly split into two main types, mural GCs (MGCs) and cumulus cells, which differentiate from each other at pre-antral to antral follicle transition. At this stage, small cavities containing follicular fluid merge to form the antrum, which separates the two cell types [24]. MGCs form the outer layer of GCs lining the follicle, while cumulus cells associate closely with the developing oocyte. Throughout pre-antral to Graafian follicular development, GCs, even those in the outer layer, maintain contact with the oocyte through by formation of filopodia known as Transzonal Processes (TZPs). Through TZPs, GCs transfer essential products of glycolysis and cholesterol metabolism, which the oocyte is unable to produce [15].

Both of the granulosa subtypes exhibit distinct gene and miRNA expression profiles, carrying out varying and specialized functions [25]. Transcriptomic analysis comparing MGCs and cumulus cells at antral stage of follicle development have shown differing roles, with cumulus cells participating in cell proliferation and metabolism, and MGCs in differentiation and cellular signaling. Important to note is that MGCs and cumulus cells continue to communicate with each other via extracellular signaling, which is vital to ovulation [22]. After oocyte release, cumulus cells accompany the oocyte while MGCs remain in the follicle [26].

## 3. The Main Metabolic and Signaling Pathways Involved in Physiological Processes of Granulosa Cells

**Figure 1:**
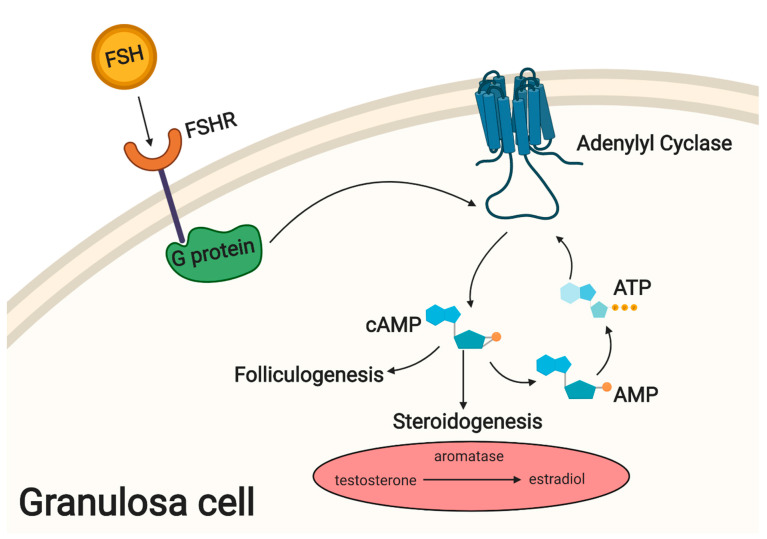
FSH & the cAMP pathway in granulosa cells.

Hormone secretion involving the Hypothalamic/Pituitary axis drives the follicular development. Gonadotropin releasing hormone (GnRH) from the hypothalamus stimulates anterior pituitary to produce and secrete FSH and LH during female puberty and reproduction [27]. GCs contain G-protein coupled FSH receptors on membrane surfaces, where incoming signals activate the cAMP pathway and cause GCs to increase conversion of testosterone to estradiol via aromatase. Expression of FSH receptor on the granulosa cell surface marks the beginning of folliculogenesis [14]. Genes activated through the cAMP pathway further increase proliferation of GCs and support this process [28]. An overview of these molecular mechanisms is presented in Figure 1.

Rising estradiol levels in the follicle promote FSH receptor expression, and thus aid a follicle to become dominant [29]. FSH and LH concentrations peak at ovulation, then remain lower throughout the luteal phase, to prevent new follicles from developing and becoming dominant [27]. Hormone levels flux throughout the menstrual cycle to control the development of the bidirectionally communicating oocyte and follicle [16].

The growing oocyte releases several growth factors which activate metabolic pathways essential for development of the oocyte in the neighboring GCs, including transforming growth factor-β (TGF-β), fibroblast growth factor (FGF) and bone morphogenetic proteins (BMPs). TGF-β released from developing oocytes interacts with receptors on the neighboring GCs, triggering pathways leading to formation of new filopodia. In response, GCs communicate via TZPs to promote oocyte growth [15].

In primary and pre-antral follicles, anti-Mullerian hormone (AMH) and inhibin alpha, two members of the TGF-β family, are released by GCs. AMH was shown to prevent FSH and LH stimulation of steroidogenesis and thus plays an inhibitory role in follicular development [29]. Inhibin is known to prevent secretion of FSH from the pituitary gland [30]. AMH could reduce follicular sensitivity to FSH signaling and thus decreases aromatase expression, playing an important role in follicular selection. Two key genes for steroidogenesis in granulosa cells, *CYP19A1* and *P450scc*, were shown to be down-regulated by the action of AMH [29]. AMH expression is lost as the follicle matures to a more advanced stage [31].

The inhibin/activin system is composed of related dimers playing opposing roles in regulation, known to be important for early folliculogenesis and GC cell proliferation. Inhibin is known to play a role in follicular selection, from dominant to surrounding smaller follicles, through paracrine mechanisms [30]. Mice with homozygous null mutations at the *inhibin a* locus demonstrated phenotypes of greatly increased size and numbers of GC layers by postnatal day 12 [31]. Although folliculogenesis was greatly accelerated, number of primordial follicles formed did not significantly increase. Notably, oocyte growth was not synchronous with increased GC development, possibly due to changing expression of growth factors important for maintaining this balance, such as GDF9, BMP15 and KITL [31]. KITLG is known to enhance oocyte growth after secretion by surrounding GCs, whereas GDF9 and BMP15 are produced by the oocyte and act on GCs to regulate their proliferation and differentiation [24].

**Figure 2:**
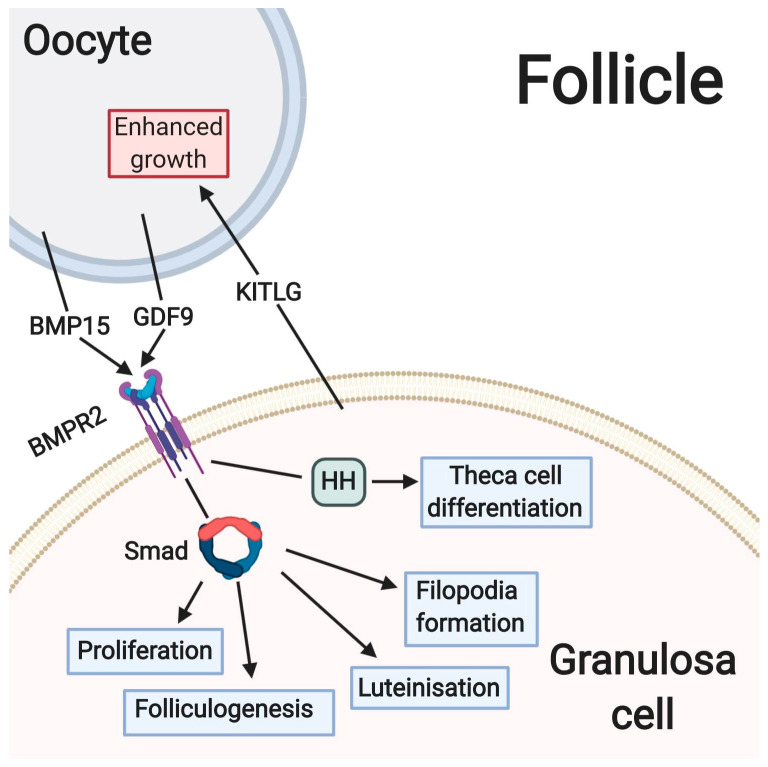
Oocyte-specific growth factors in folliculogenesis.

At the pre-antral stage of folliculogenesis, GDF9 from the oocyte causes neighboring GCs to initiate hedgehog signaling, culminating in differentiation of theca cell precursors to form the theca layer. Expression of Hh ligand in GCs is regulated by GDF9. The expression of Gli1 marks the differentiation of theca cells, a process which does not happen in the absence of Ihh and Dhh signaling [32]. Both GDF9 and BMP15 bind to type II BMP protein receptors in granulosa cells and thus initiate intracellular SMAD signaling. This BMP-SMAD1/5/8 pathway is important in follicular activation and development, GC cell proliferation, atresia and luteinization [28]. A summary of these processes is presented in Figure 2.

It has been hypothesized that the BMP-SMAD1/5/8 pathway is functionally linked to the gonadotropin/cAMP pathway in developing GCs [28]. FSH was shown to upregulate the expression of various BMP receptors, as well as enhance SMAD signaling in the granulosa-like KGN tumor cell line [33]. In the same cell line, BMPs downregulated FSH cell surface receptor expression. Additionally, GDF9 reduced steroidogenesis and LH receptor expression in rat granulosa cells [33]. Consequently, it appears that a complex system of regulation controlling signaling pathways governs follicular and oocyte development.

The Notch pathway in GCs originates from gonadotropin signals and is important for oocyte development. Kinase cascade activation by the Jag1 ligand promotes GC differentiation and inhibits proliferation [34].

**Figure 3:**
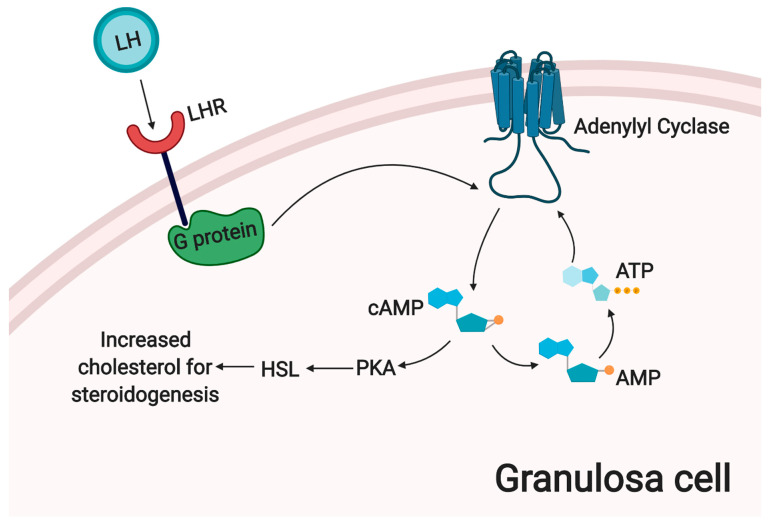
Steroidogenesis in the corpus luteum.

Steroidogenesis in the adult ovary is a complex, multi-level process. In the pre-antral follicles, following differentiation of theca cell precursors, these cells convert cholesterol to androgens. In turn, neighboring GCs aromatize these androgens to estrogens. In this way, hormone production is greatly increased as folliculogenesis progresses. Luteinized GCs express steroidogenic enzymes inducing production of progesterone instead of estradiol, and further increasing steroid production overall, a process which requires higher total amounts of cholesterol. Most of the required cholesterol for late stage steroidogenesis is derived from HDL and LDL in the bloodstream [35]. LH signals in theca lutein cells (small lutein cells) trigger an increase in intracellular cAMP levels, which could lead to increased bioavailability of cholesterol via activation of Protein Kinase A (PKA) and Hormone Sensitive Lipase (HSL) [35]. HSL can release cholesterol from storage in form of lipid droplets. Progesterone production is inhibited by activation of the AMP-activated protein kinase (AMPK) pathway in rat granulosa cells, possibly through the reduced expression of key enzymes such as 3βHSD, P450scc and StAR [26]. The process of corpus luteum steroidogenesis is summarized in Figure 3.

Together with theca cells, GCs luteinize to form the corpus luteum following the rupture of the follicle, a process associated with rapid vascularization. The key to this process is the uncoupling of the complex interdependent relationship of the oocyte with surrounding GCs [19]. This point marks terminal differentiation of GCs and their subsequent exit from the cell cycle. Although removal of the oocyte from the follicle can trigger luteinization, high levels of LH have been also been shown to induce this process, even if the oocyte is still present. This suggests a complex array of regulation in GC differentiation [23].

Hippo signaling is known to play important roles in maintaining animal organ sizes by controlling regulation of cell proliferation. In folliculogenesis, hippo signaling negatively regulates the release of growth factors by phosphorylation of the main signaling protein, YES-Associated protein 1 (YAP) [36].

Insulin-like growth factor 2 (IGF-2) works alongside FSH and is essential for granulosa cell differentiation, proliferation and steroidogenesis in humans. In fact, GCs cannot differentiate without stimulation by FSH alongside IGF-2 [37]. Expression of IGF-2 in GCs is enhanced by FSH in an AKT-dependent manner. The dependency of FSH on IGF-2 and vice versa in GC processes could play a role in follicular selection [37]. A recent study suggested that interaction of GDF9 and BMP15 with FSH further potentiates expression of IGF-2 in human granulosa cells. Additionally, aromatase is regulated by FSH and BMP15/GDF9 [38]. However, the exact mechanisms of interactions between oocyte-derived growth factors BMP15, GDF9, FSH and IGF-2 are yet to be fully investigated.

Finally, the phosphatidylinositol 3 kinase (PI3K) pathway is known to be linked to follicular activation in species such as rat and mouse. Small, naturally occurring molecules such as resveratrol may regulate activation of the PI3K pathway and thereby affect follicle activation and maturation of pre-granulosa cells [8]. Inhibitors of the PI3K pathway may inhibit action of aromatase, reduce expression of key genes such as LHR, as well as prevent IGF-1 and FSH co-activity [39].

## 4. Stemness of Human Ovarian Granulosa Cells

GCs have been experimentally shown to be a multipotent cell population [7,14]. It has been suggested that it is made up of cells at various stages of differentiation, thereby showing different levels of stemness. The stem-like population may express FSHR but not LHR. As expression of LHR increases, these cells closer resemble GC phenotype, with expression of FSHR and LHR during luteinization, with the physiological characteristics progressively fading during the course of in vitro culture [7].

Along with gradual loss of GC function markers i.e., FSHR, aromatase, throughout the time of their in vitro culture, the expression of markers characteristic for mesenchymal stem cells (MSC) phenotype increases. GCs were successfully differentiated into a variety of cell types, including neurons, osteoblasts and chondrocytes [40]. GCs also undergo cell division whilst in contact with neighboring cells, without the presence of substratum, a property normally only seen in a stem cell phenotype [13]. In vitro culture of GCs was also characterized by the formation of basal lamina, suggesting that the follicular basal lamina is formed by granulosa cells [13]. Furthermore, both granulosa and theca cells spontaneously luteinize in vitro [23]. Granulosa cells share some similarities with cells of epithelial origin, secreting basal lamina and containing adherens junctions, believed to be important for follicle growth initiation. However, they do not exhibit junctions or desmosomes, as well as they lack any epithelial cell markers [18].

Granulosa cells express vimentin [18], while luteinized GC populations were also reported to express Oct-4 [14,40], further reinforcing suggestions of resemblance to MSC populations. Oct-4 is one of the main regulatory proteins in cell differentiation and self-renewal. Therefore, granulosa cells have some characteristics of epithelial cells but are more accurately defined as of stem cell/mesenchymal phenotype. When it comes to ovary associated MSCs, small embryonic stem cells (VSELs) constitute a quiescent and multipotent population, whereas ovarian stem cells (OSCs) are a larger and actively dividing mesenchymal population, both found in the epithelial layer. VSELs, expressing embryonic markers such as Oct-4, are found embedded in the ovary surface epithelium (OSE) [41]. Both VSELs and OSCs participate in formation of the oocytes and primordial follicles in response to FSH. Formation of the primordial follicle is also preceded by epithelial-mesenchymal transition of epithelial cells lining the surface of the ovary [41]. With the onset of ovulation, the basal lamina is ruptured, and the inner layer of GCs become more loosely packed, less polarized, and increasingly proliferative, all being characteristics of the mesenchymal cell type [18]. The MGCs greatly increase in size to form luteinized cells. This process represents yet another example of epithelial-mesenchymal transition in adult tissue, a rare occurrence in the physiological conditions of the adult human body [26].

Immediately before the rupture of the follicle, GCs shift their steroid production from 17-β-estradiol to progesterone. After ovulation, GCs, remaining in contact with endothelial cells and producing extracellular matrix, undergo hypertrophy to differentiate into large luteal cells. Simultaneously, centripetal angiogenesis occurs, starting from the vascular network surrounding the follicle, while the follicular basement membrane is destroyed and endothelial cells migrate into the inner GCs layer.

Blood platelets have been suggested as the regulators of endothelial cell migration and granulosa cell luteinization during development of the human corpus luteum [42]. Accordingly, Basini et al. have shown that platelet lysate stimulates the luteinization of swine granulosa cells since it was observed to convert estradiol to progesterone. Platelets, containing hemoattractive substances, have also been demonstrated to induce angiogenesis [43]. It supports the hypothesis that platelets would be useful tool in regenerative medicine. Furthermore, the results of Basini et al. could be of interest as ovarian pathologies are often related to the dysfunctions of platelets.

Again, the expression of stem cell markers, such as Oct-4, Nanog and Sox-2, in isolated GCs has been shown experimentally. However, marker expression varied between the investigated species, as well as with the maturation stage of isolated granulosa cells. For example, Oct-4 expression was detected in human but not in porcine model immediately after isolation of GCs [44,45]. While this fact is one of the many indicators that the stemness of granulosa cells needs to be further investigated to become a consensus, it is certainly a population of high potential for possible applications in the fields of modern medicine. Hence, studies of compounds able to evoke specific processes in GCs, especially related to their induced differentiation into different lineages are crucial to determine the usefulness of their in vitro cultures as experimental models and basis for cell-based therapies. Resveratrol is one of such potential molecules.

## 5. Resveratrol—Chemical Structure and Biological Effects

Resveratrol is a well-known phytoalexin, synthesized by plants as a response to stressful environmental factors, such as microbial and fungal infections, ultraviolet radiation, injuries, temperature fluctuations, and ozone exposure [3,4,46]. Additionally, Hain et al. demonstrated increased disease resistance of transgenic plants, which can express foreign genes coding for phytoalexins [47].

**Figure 4:**
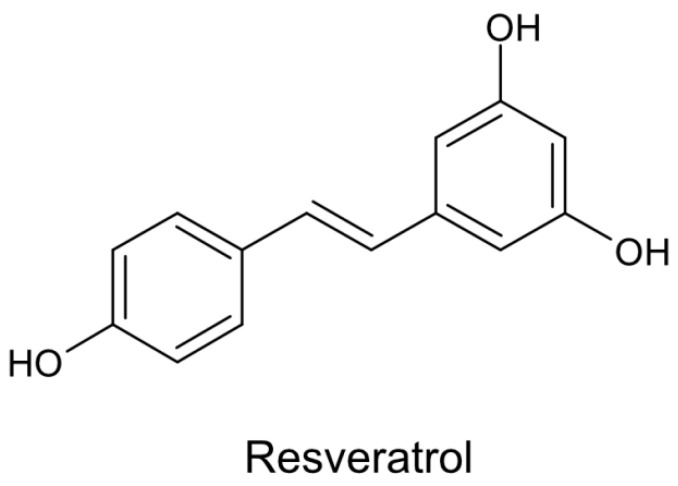
Chemical structure of trans-resveratrol.

The stilbene-based scaffold of resveratrol composed of two phenolic rings occurs in two isoforms, cis and trans. Generally, trans-stilbene analogs are less active than matching cis-isomers except for trans-resveratrol (Figure 4), which exerts more potent activity and is the main isoform prevalent in plants [48,49].

Resveratrol has been found to exert a wide spectrum of biological activities, including estrogenic, anti-inflammatory, antioxidant, antifungal, neuro- and cardioprotective ones.

Health benefits provided by resveratrol have been reported in a dose-dependent manner. At a lower dose, resveratrol displays antiapoptotic and cardioprotective properties, evidenced by improved post-ischemic ventricular recovery and reduction of myocardial infarction size. In contrary, at a higher dose, resveratrol induces apoptosis in cancer cells and depresses cardiac function [50].

Although the anti-tumor effect of resveratrol became an area of intense scientific research, its positive biological effects in other disease models have been also revealed (Table 1).

## 6. Resveratrol as a Pro-Differentiation Agent

To date, many studies showed essential roles of several signaling pathways and molecules, such as mTOR, AKT or AMPK, in promotion of cell differentiation [71]. Resveratrol has been revealed to induce differentiation of MSCs into various specific cell types. As was mentioned in the previous chapters, while there is no consensus that ovarian granulosa are a population of MSCs, they certainly present a range of mesenchymal-like characteristics. One of the most promising properties of GCs is the ability for differentiation into several lineages of mesenchymal origin, which strongly supports their link to MSCs and is a basis of the studies on the effect of resveratrol on their in vitro cultures.

Peltz et al. provided an insight into the way in which resveratrol regulates self-renewal and differentiation of human MSCs derived from adipose tissue, indicating its biphasic effect. After a short- and long-term exposure, resveratrol has been reported to enhance self-renewal and osteogenic differentiation, while promoting adipogenesis only after a long-term exposure. Moreover, the osteogenic and adipogenic differentiation was related to the dose of resveratrol during treatment [72]. These results are consistent with other authors’ findings, in which resveratrol promoted osteoblast differentiation of MSCs via SIRT-1 activation [73] and ER/NO/cGMP pathway [74]. Dai et al. have also described the enhancing effect of resveratrol on proliferation and differentiation of human bone-marrow MSCs into osteoblasts. Furthermore, the MAPK signaling pathway has been shown to be essential in resveratrol- mediated anabolic activity in cells [75].

Resveratrol has been revealed to induce differentiation of vascular smooth muscle cells in dose-dependent manner, as different doses of this compound exerted activity via specific mechanisms. Low concentration of resveratrol has been shown to activate SIRT-1 and consequently stimulate differentiation through AKT activation. In turn, its high concentration provoked differentiation via AMPK-mediated inhibition of mTOR pathway, also initiating AKT [71]. In addition, Zhao et al. indicated that resveratrol has the ability to enhance differentiation of canine bone marrow mesenchymal stem cells into osteoblasts in a different way; via Wnt/beta-catenin and ERK/MAPK signaling pathways activation [76].

Resveratrol has also been found to promote neural differentiation of human umbilical cord derived MSCs in a dose-dependent manner. Wang et al. revealed that resveratrol treatment caused morphological alterations of cells and changes in expression of neural markers, such as Nestin, βIII-tubulin and NSE as well as pro-neural transcriptional factors, like Neurogenins (Ngn1, Ngn2) and Mash1. Moreover, resveratrol was reported to enhance cell viability and proliferation via promotion of SIRT-1 and proliferating cell nuclear antigen (PCNA) expression, while decreasing the expression of p53 and p16 [77]. Similarly, Gou et al. confirmed that resveratrol treatment induces differentiation of human umbilical cord derived MSCs into neuron-like cells, evidenced by increased levels of Nestin and NSE protein, but only at concentration of 15 mg/L and higher [78].

Interestingly, resveratrol was reported to significantly stimulate 3T3-L1 adipocyte culture differentiation at physiologically achievable low doses (1 and 10 μM), while previous studies showed that the higher concentration ranges of 20–100 μM, less possible to achieve in vivo, exerted suppressive effects on cells [79].

Recent studies indicated that polydatin, glycosylated precursor of resveratrol, exerts pro-differentiation activity in MSCs. Di Benedetto et al. revealed that polydatin promotes MSCs osteogenic differentiation from dental tissue and improves mineral matrix deposition. Due to its better metabolic stability and pharmacokinetic properties than resveratrol, polydatin has been suggested as a potential non-pharmacological therapy ameliorating bone health [12].

## 7. Resveratrol as a SIRT-1 Activator in GCs

Sirtuins are a family of nicotinamide adenine dinucleotide-dependent proteins with deacetylase and/or mono-ADP-ribosyltransferase activity. They are involved in certain important cellular processes, such as maintaining genomic stability or transcriptional gene silencing, and are considered as the main antiaging molecules [80,81]. Seven members of the sirtuin (SIRT 1-7) family have been described in mammals so far, presenting specific localization, function and substrate complementarity [82]. SIRT-1 is the closest mammalian homolog of yeast Sir2 (silent information regulation 2), which was found to block the transcription of FoxO1 and FoxO3, and target the p53, PGC-1α, and hypoxia-inducible factor 2α (HIF2α) molecules [80,83]. Recent studies demonstrated that SIRT-1 is also a molecule playing a pivotal role in controlling ovarian function. The expression of SIRT-1 has been observed at numerous stages of folliculogenesis, in nuclei of GCs, as well as in theca cells and the oocytes [84]. Han et al. evaluated the expression of SIRT-1 and the induction of SIRT-1-mediated apoptosis in human GCs. Their results demonstrated differences in levels of SIRT-1 expression in GCs in patients with distinct ovarian pathologies; the SIRT-1 protein level in GCs from poor ovarian response patients was significantly lower compared to healthy and PCOS patients. Additionally, it has been suggested that SIRT-1 prevents GCs apoptosis through ERK1/2 signaling pathway activation, widely observed in ovarian cells [85]. Moreover, deacetylation of FOXL2 by SIRT-1 in KGN and COV434 human granulosa cell lines has been found to be essential to maintain cell homeostasis [86]. Pavlova et al. revealed that SIRT-1 stimulates cell proliferation by increasing accumulation of cyclin B1 and cyclin-dependent protein kinase Cdc2/p34 in porcine GCs. Furthermore, the results showed the interrelation of SIRT-1 and NF-κB. The balance between these two molecules has been suggested to be extremely important in proliferation processes and secretion activity of porcine GCs [81,87].

**Figure 5:**
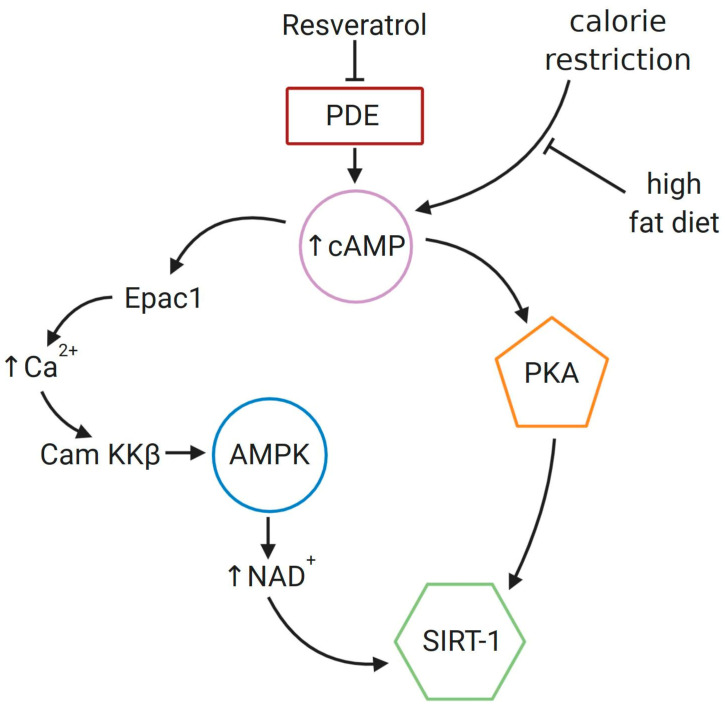
Mechanism of SIRT-1 activation by resveratrol in GCs. Resveratrol inhibits activity of cyclic nucleotides phosphodiesterase (PDE), which results in an increase of 3′,5′-cyclic adenosine monophosphate (cAMP). In turn, activation of the exchange guanine nucleotide Epac1 occurs, followed by enhanced level of intracellular calcium. Ca^2+^/CaM-dependent protein kinase kinase β (CaMKKβ) activates AMPK through phosphorylation, which causes an increase in the level of NAD^+^ and, in consequence, activation of SIRT-1. The second suggested mechanism of SIRT-1 activation is the phosphorylation by protein kinase PKA, activated by higher cAMP level [91,92].

Resveratrol has been identified as a potent indirect SIRT-1 activator (Figure 5). Several studies have shown partial ability of resveratrol to mimic calorie restriction through SIRT-1 activation. Moreover, as a consequence of sirtuin deacetylase activation, resveratrol has been suggested to extend lower organisms’ lifespan [2,80]. Previously conducted studies revealed that resveratrol remarkably enhances binding affinity of a substrate to SIRT-1 by reducing its Michaelis constant (Km) value, but without an effect on turnover number of the enzyme [88].

On the other hand, Hou et al. provided new insights into the mechanism by which resveratrol activates the SIRT-1 pathway in GCs. Resveratrol has been found to stabilize SIRT-1-peptide interactions in a substrate specific manner. In addition, the authors indicated the fundamental role of N-terminal domain in recognition of the substrate [89].

Interestingly, after in vitro fertilization, the beneficial impact of resveratrol treatment has been shown on bovine oocyte maturation and embryo development, as this compound induced progesterone secretion and exerted antioxidant activity. These beneficial effects of resveratrol have been suggested to be associated with SIRT-1 activation [90].

## 8. The Modulation of Granulosa Cell Physiological Processes by Resveratrol

Since resveratrol has been reported to exert wide spectrum of beneficial effects on health through several mechanisms, including anti-oxidative, anti-inflammatory and anti-aging, its application in ovarian dysfunction treatment has also started to be considered [2]. Recent extensive research has suggested that resveratrol might improve ovarian function and indicated this polyphenol compound as a regulator of ovarian development. Resveratrol has been revealed to promote growth of human ovarian follicles in in vitro culture system. Studies performed in animal models confirmed these observations since resveratrol induces healthy follicles development and protects them from atresia [93,94,95]. To date, mechanisms of resveratrol action on follicle growth are still elusive. However, resveratrol has been suggested to be involved in sirtuin activation, estrogen and mTOR signaling pathways, telomere elongation or AhR activity [2,93,94].

### 8.1. Effects of Resveratrol on Ovarian and Follicular Function

Recent studies revealed that resveratrol promotes growth of human ovarian follicles in tissue culture model [93]. Banu et al. demonstrated that resveratrol, after toxic chromium exposure, decreases the DNA fragmentation related to apoptosis in oocytes and granulosa cells [96]. These findings stay in accordance with results of Bazerra et al., reporting that resveratrol enhances primordial follicle activation and decreases the level of DNA fragmentation in in vitro cultures of ovine ovarian tissue. Furthermore, they have also shown the ability of resveratrol to stimulate GCs proliferation via activation of the PI3K pathway [44,97]. PI3K is known as one of the fundamental signaling pathways, involved in follicle activation in rodents [44]. Interestingly, in cancer cell lines, resveratrol has been reported to down-regulate the PI3K/AKT pathway, as well as induce apoptosis and exert chemotherapeutic activity [98]. Morita et al. demonstrated that resveratrol also inhibits viability of GCs, without causing their apoptosis [84]. Concomitantly, another study on human granulosa cell lines revealed that resveratrol diminishes apoptosis and causes inhibition of caspase-3. Subsequently, the authors suggest that resveratrol increases the phosphorylation of ERK1/2 in COV343 granulosa cell line and human luteinized GCs [85]. These results are in agreement with findings of Ortega et al., since resveratrol at higher dose has been shown to inhibit DNA synthesis in rat ovarian GCs and to exert cytostatic but not cytotoxic effect. Concomitantly, low concentration of resveratrol stimulated thymidine incorporation [99]. Summarizing, these findings suggest that resveratrol, through activation of SIRT-1 and ERK1/2 signaling pathways, might inhibit apoptosis in the ovarian GCs. Additionally, Lee et al. revealed that resveratrol enhances porcine oocyte in vitro maturation, cumulus cells expansion and, subsequently, embryo development via sonic hedgehog signaling pathway [100]. Interestingly, Wong et al. demonstrated the ability of resveratrol to inhibit cell proliferation and induce a concentration-dependent induction of apoptosis in rat ovarian theca-interstitial cells, which are crucial to modulation of the ovarian function [101]. Different effects of resveratrol in theca and granulosa cells suggest that this compound may be a potential treatment in infertility induced by obesity and polycystic ovarian syndrome (PCOS), through maintenance of the balance between these two cellular compartments [99,102]. PCOS is an endocrine disorder frequently associated with oxidative stress and persistent low-grade inflammation. Moreover, PCOS is considered as one of the major causes of infertility in reproductive-aged women [103]. In the first randomized, double-blind placebo-controlled study Banaszewska et al. evaluated resveratrol (1500 mg p.o.) therapy in young PCOS patients over a period of 3 months. They observed a significant decrease in total testosterone, DHEAS as well as in fasting glucose in comparison to control [104]. The latest study of Brenjian et al. confirmed beneficial effect of resveratrol treatment in women with PCOS. Resveratrol, through its anti-inflammatory action, has been found to decrease the serum levels of pro-inflammatory factors such as interleukins (IL-6, IL-1β, IL-18), tumor necrosis factor (TNF-α) and C-reactive protein (CRP) [105]. Several studies indicated CRP as a fundamental inflammatory marker, the level of which is significantly elevated in PCOS [106,107]. Resveratrol has also been reported to decrease NF-κB protein level and alter endoplasmic reticulum stress in GCs by modulating the expression of genes related to unfolded protein response [105]. Furthermore, the previous studies revealed promising outcomes of combined treatment with metformin and resveratrol in in vivo rat model of PCOS. Moreover, resveratrol has been indicated as a potential alternative to metformin treatment, since this phytoalexin exerts antioxidant and anti-inflammatory effects associated with SIRT-1 and AMPK induction [103]. Beneficial activities of resveratrol in PCOS patients have been also confirmed in randomized clinical trials, results of which revealed significantly lower expression of VEGF and HIF1 in the angiogenesis pathway of GCs after 40 days of administration [108]. Based on these findings, resveratrol might be proposed as a potential additional therapy for women with PCOS.

### 8.2. Anti-Oxidant and Estrogenic Activity of Resveratrol

Oxidative stress has been found to play a crucial role in obesity-associated infertility. Oxidized low-density lipoprotein (oxLDL) level is elevated around 2-fold in obese woman, with its higher concentration in preovulatory follicles negatively impacting in vitro fertilization outcomes [109]. Presence of oxLDL receptors on GCs was previously demonstrated, with recent studies showing that their activation by oxLDL leads to survival autophagy and apoptosis in human GCs, thus determining the possible mechanism of infertility [102,110]. Shube et al. showed that resveratrol protects different subtypes of GCs from degeneration through several mechanisms, such as diminishing the oxidative stress, decreasing expression of the oxLDL-binding receptors (LOX-1, TLR4, CD36) as well as heat shock protein 60 synthesis and mitosis enhancement [102].

PI3K/Akt/mTOR signaling pathway is known to play a key role in the oocyte growth, as well as proliferation and differentiation of GCs. Accordingly, resveratrol has been reported to reduce oxidative stress in rat model of premature ovarian insufficiency by inducing the expression of factors involved in PI3K/Akt/mTOR pathway activation [97].

Over the last years, phytoestrogens, as naturally occurring plant compounds, have been in the center of many researchers’ interest due to their estrogen receptor (ER) agonistic or antagonistic activity and potential clinical application in many hormone-related disorders. Phytoestrogens have been previously suggested as plant alternative for postmenopausal hormone therapy.

Study of Sharma et al. described the expression and function of ER in rat GCs. ERβ subtype has been revealed to be the predominant steroid receptor in GCs, whereas the expression of ERα is present at a significantly lower level [111]. Due to the fact that resveratrol, as a phytoestrogen, binds to ERs α and β equally and is structurally similar to estrogens, its steroid activity is now of the high interest. To date, it has been revealed that resveratrol inhibits steroidogenesis in hormone-producing cells, such as rat Leydig and adrenocortical cells [80]. Morita et al. reported that after resveratrol treatment, the mRNA level of SIRT-1, LH receptors, steroidogenic acute regulatory protein (StAR) and P450 aromatase were significantly increased in rat granulosa cells. Concomitantly, the level of FSH receptor was unaffected. Resveratrol was also shown to induce 3-fold enhancement of progesterone secretion in rat GCs [84]. The up-regulation of StAR was also reported in KGN human granulosa-like tumor cells [112]. StAR is known to play pivotal role in the steroidogenesis process, which is related to the initiation of steroidogenesis through delivery of cholesterol from cytosol to the mitochondria [113,114]. It is also crucial for the corpus luteum development and maintenance. Consequently, the corpus luteum, developed from remaining granulosa and theca cells, produces progesterone, which is required for embryo implantation and normal development of pregnancy [84,115]. On the other hand, Ortega et al. reported that resveratrol diminished estrogen secretion in rat granulosa cells [99].

## 9. The Activity of Resveratrol Derivatives

Resveratrol has been shown to be more effective when administered topically, rather than orally due to its extensive metabolism and rapid excretion [116]. Although the oral absorption of resveratrol is high and reaches about 75%, extensive liver and intestine metabolism results in bioavailability substantially lower than 1%. Several studies have revealed that major metabolites of resveratrol are glucuronides and sulfate conjugates. Hence, the therapeutic application of resveratrol is limited due to poor bioavailability and fast elimination from the circulation [117,118]. Its structural alteration to obtain analogs with improved pharmacokinetics and stability are currently of high significance. The structure-activity relationship studies have revealed that methoxy substitution of hydroxyl groups increases the stability of the molecule by diminishing the susceptibility to phase II conjugation reactions in vivo. Therefore, resveratrol analogs (e.g., methylated ones) with improved pharmacokinetics may be a solution to the problem of low bioavailability. Accordingly, Basini et al. demonstrated differences in activities among resveratrol analogs, polymethoxystilbenes: 3,5,4′-trimethoxystilbene and 2-hydroxy-3,5,4′-trimethoxystilbene, in porcine GC model. The 2-hydroxy analog showed a more potent inhibitory effect on GC proliferation than the trimethoxystilbene derivative. However, both tested compounds inhibited VEGF production and stimulated steroidogenesis [119]. The inhibitory effect on VEGF mRNA and protein expression was also observed in rat granulosa cells [99].

## 10. Conclusions

In the light of these findings, resveratrol and its analogs might be considered as the potential therapeutic agents in various ovarian disorders. It has been revealed that resveratrol protects granulosa cells from degeneration, and simultaneously enhances proliferation and differentiation of GCs. Resveratrol has also been found to modulate ovarian function, as well as promote oocyte growth, maturation and embryo development. In addition, the administration of resveratrol may be of clinical relevance due to its anti-inflammatory phytoestrogenic activity.

Resveratrol might be relevant in treatment of conditions associated with increased vascular permeability and VEGF overexpression, due to its VEGF expression inhibiting activity. Since resveratrol has been reported to adjust the ratio of theca to granulosa cells, the recent studies suggested its therapeutic potential in PCOS, the condition associated with thecal hyperplasia, increased vascularization of ovarian tissue and alternation in function and proliferation of theca and GCs.

Several studies showed beneficial effects of resveratrol treatment on GC functions, while there is only one study presenting how resveratrol derivatives impact human ovarian GCs proliferation and secretion profile. These kinds of studies seem to be especially important nowadays, as GCs share many MSC characteristics, and hence might have potential applications as a research model and a basis of cell-based therapies. Since resveratrol analogs have been reported to be well absorbed and exerted a wide spectrum of activities, not only in vitro but also in vivo, further investigations of their activity on human ovarian GCs are certainly necessary.

## Figures and Tables

**Table 1:** The activities of resveratrol in selected morbidities.

Condition	Effects of Resveratrol Administration	References
**Alzheimer’s disease**	protective effects in Aβ1-42-treated PC12 cells via reduction of apoptosis and reduction of oxidative status and mitochondrial damage	[51]
	anti-inflammatory effect via suppression of M1 microglia activation, enhancement of Th2 responses and promotion of anti-inflammatory cytokine and SIRT-1 expression	[52]
	reduction of NF-κB signaling in microglia, which plays a pivotal role in Aβ- dependent neurodegeneration via SIRT-1 activation	[53]
**Parkinson’s disease**	inhibition of α-synuclein aggregation, reduction of the total α-synuclein and oligomers levels and decrease in cytotoxicity, neuroinflammation and oxidative stress in the A53T α-synuclein mouse model of PD in a dose-dependent manner	[54]
	modulation of the MALAT1/miR-129/SNCA signaling pathway via an increase in TH+ cell number and miR-129 expression and decrease in expression of SNCA and MALAT1 by blocking the transcription of its promoter	[55]
	neuroprotective effect through mitochondria dynamics modulation and upregulation of autophagic flux associated with MEK/extracellular signal-regulated kinase signaling pathway	[56]
**Huntington’s disease**	significant improvement of motor coordination and learning through enhancement of expression of mitochondrial-encoded electron transport chain genes in YAC128 mice, related to increased activation of SIRT-1	[57]
**Depression**	antidepressant- like effects in mice via decrease of immobility time in the forced swim test and tail suspension test without affecting locomotor activity in the open field test, lowering serum corticosterone level and increasing brain-derived neurotrophic factor (BDNF) protein and extracellular signal-regulated kinase (ERK) phosphorylation level	[58]
	reversion of the chronic unpredictable mild stress- induced behavioral abnormalities and biochemical changes and normalization of phosphorylation of Akt and mTOR in the hippocampus prefrontal cortex	[59]
**Pain**	peripheral antinociceptive effect related to potassium channel activation	[60]
	antinociception after local application in formalin test	[61]
**Diabetes mellitus**	exercise-like effects in patients with type 2 diabetes mellitus via energy expenditure regulation, associated with increased SIRT-1 and AMPK expression in skeletal muscle	[62]
	increase of PDPK1, mTOR and FOXO1 expression in insulin resistant HepG2 cells, affecting insulin resistance	[63]
	enhancement of insulin sensitivity, lowering blood sugar level, simultaneously reducing resistin expression in rats with diabetes	[64]
	reduction of insulin resistance and, in consequence, decrease of blood sugar level via Akt pathway activation in male patients with type 2 diabetes during a randomized controlled study	[65]
	reduction of fasting blood glucose and HbA1c level in type 2 diabetes patients	[66]
**Obesity**	reduction of high-fat diet induced obesity in mice in a dose-dependent manner; potentiation of cytotoxicity and suppression of adipogenesis in 3T3-L1 cells and inhibition of lipolysis in mature adipocytes	[67]
	reduction of post-prandial hyperglycemia via inhibition of intestinal α-glucosidase	[68]
	promotion of more beneficial microbial profile, regulation of the production of appetite hormones and improvement in integrity of the intestinal epithelium	[69]
	reduction of adipocyte size, evidenced by a decrease in large and very-large adipocyte level and an increase in small adipocytes in obese men	[70]
